# Papillomavirus infection and male infertility: A systematic review and meta‐analysis

**DOI:** 10.1002/hsr2.70048

**Published:** 2024-08-29

**Authors:** Andrea Garolla, Silvia Mereu, Damiano Pizzol, Dong Keon Yon, Masoud Rahmati, Pinar Soysal, Petre Cristian Ilie, Alessandro Bertoldo, Mike Trott, Lee Smith

**Affiliations:** ^1^ Unit of Andrology and Reproductive Medicine, Department of Medicine University of Padova Padova Italy; ^2^ Health Unit, Eni San Donato Milanese Italy; ^3^ Center for Digital Health, Medical Science Research Institute, Kyung Hee University Medical Center Kyung Hee University College of Medicine Seoul South Korea; ^4^ Department of Physical Education and Sport Sciences, Faculty of Literature and Human Sciences Lorestan University Khoramabad Iran; ^5^ Department of Physical Education and Sport Sciences, Faculty of Literature and Humanities Vali‐E‐Asr University of Rafsanjan Rafsanjan Iran; ^6^ Department of Geriatric Medicine, Faculty of Medicine Bezmialem Vakif University Istanbul Turkey; ^7^ The Queen Elizabeth Hospital Foundation Trust King's Lynn UK; ^8^ Unit of ART “G.Beltrame”, Department of Obtetrics and Ginecology, Ospedale di Oderzo Treviso ULSS2 Italy; ^9^ Princess Alexandra Hospital University of Queensland Building 33 Brisbane Queensland Australia; ^10^ Metro South Addiction and Mental Health Services Brisbane Queensland Australia; ^11^ Centre for Health, Performance and Wellbeing Anglia Ruskin University Cambridge UK

**Keywords:** sperm quality, sperm parameters, male infertility, HPV

## Abstract

**Background and Aims:**

Increasing attention is being paid to the role of human papillomavirus (HPV) in men and specifically reproduction. Growing evidence suggests an association between HPV infection with many adverse effects including the impairment of semen parameters, the increase of blastocyst apoptosis, the reduction of endometrial implantation of trophoblastic cells, as well as the increase rate of miscarriages and spontaneous preterm birth.

**Methods:**

We systematically searched PubMed/MEDLINE, Scopus, Embase, Web of Science, CINHAL, PsycINFO, and ERIC from inception to 2nd of July 2024, for studies that investigated the association between HPV infection with sperm parameters and fertility outcomes. The meta‐analysis was conducted on mean data and standard deviations.

**Results:**

We included 25 studies with a total of 6942 patients. Sperm morphology was lower in HPV positive groups versus HPV negative control groups (SMD = ‐0.52 95% CI −0.84; −0.21; *p* = 0.001). Sperm motility was also significantly lower in HPV positive groups when compared to HPV negative controls (SMD = −0.82 95% CI −1.07; −0.57; *p* = <0.001). Sperm volume, concentration, and pH were not significantly different between the two groups. The other 15 studies included in the systematic review for which it was not possible to conduct a meta‐analysis showed strong associations between HPV infection and impairment of sperm parameters, reduced couple fertility and increased risk of pregnancy loss.

**Conclusions:**

The current evidence highlights the link between HPV infection and sperm parameters, male fertility and reproductive outcomes, which has the potential to lead to a decreased couple fertility, increased risk of pregnancy loss, re‐infection and increased treatment costs.

## 1 INTRODUCTION

Human papillomavirus (HPV) is the most common sexually transmitted viral infection worldwide affecting both males and females.^1^ There are more than 200 known subtypes including High‐Risk‐HPV (HR‐HPV) and Low‐Risk HPV based on their oncogenic potential.^2^ It has been shown that most sexually active men and women acquire at least one HPV infection at some point in their lives and, some of them, could be repeatedly infected although most infections remain asymptomatic.^3^ Interestingly, the global prevalence of HPV infection in women with normal cytology is around 11–12%, with the highest prevalence in sub‐Saharan Africa at 24%, Eastern Europe 21% and Latin America 16%. Moreover, HPV prevalence peaks in adolescence and those under 25 years old.^4^ Although HPV infection in men is less investigated than in woman, the global prevalence in men is estimated to be ~30% with the infection peak between the ages of 25 and 29 years and a HR‐HPV prevalence of 21%.^1^ In general, HPV clinical manifestation in men includes anogenital warts and penile, anal, and oropharyngeal cancers and recurrent respiratory papillomatosis.^5^, ^6^ For women, The World Health Organization (WHO) guidelines recommend using HPV DNA detection every 5–10 years as a primary screening test for those aged between 30 and 50 years and, when HPV DNA is not available, visual inspection after acetic acid application or cytology every 3 years.^7^ Although sexually active men are at risk of HPV‐related morbidity and represent a reservoir for HPV, there is no standardized approach for screening in men.^8^ However, increasing attention is being paid to the role of HPV in men also due to its role in reproduction. Indeed, growing evidence suggests associations between HPV infection with multiple adverse effects including the impairment of semen parameters, the increase of blastocyst apoptosis, the reduction of endometrial implantation of trophoblastic cells, the increase rate of miscarriages, and spontaneous preterm birth.^9^ Interestingly, men affected by idiopathic infertility show a higher prevalence of HPV infection, asthenozoospermia and anti‐sperm antibodies (ASAs) compared with the general population.^10^ Moreover, HPV in semen has been demonstrated to also have an impact on reproductive outcomes.^11^ To date, the role of HPV in male and couple fertility it is not fully understood and the scientific literature addressing male aspects remains less documented than for females. The aim of this review and meta‐analysis was to assess associations between HPV infection with sperm parameters and its correlation with couple fertility.

## 2 METHODS

This systematic review adhered to the PRISMA^12^ and MOOSE^13^ statements and followed a structured protocol registered on PROSPERO (CRD42024510030).

### 2.1 Data sources and literature search strategy

Two investigators (MT and DP) independently conducted a literature search using PubMed/MEDLINE, Scopus, CINAHL, Embase, PsycINFO, Web of Science and ERIC from inception until July 2nd, 2024. Any inconsistencies were resolved by consensus with a third author (LS).

In PubMed, the following search strategy was used: (“Human Papillomavirus” OR “Papillomaviridae” OR HPV) AND (“male fertil*” OR “male infertil*” OR “male subfertil*” OR “fertil* men” OR “infertil* men” OR “subfertil* men” OR “Sperm” OR “Sperm quality” OR “Sperm count” OR “Sperm volume” OR “Sperm motility” OR “Sperm vitality” OR “Sperm antibodies” OR “Sperm pH” OR “Sperm viscosity” OR “Sperm morphology” OR “Sperm DNA” OR “sperm DNA fragmentation” OR “Sperm DNA integrity” OR “semen quality” OR “semen parameters”). Conference abstracts and reference lists of included articles were hand‐searched to identify any potential additional relevant work.

### 2.2 Study selection

Following the PICOS (participants, intervention, controls, outcomes, study design) criteria, we included studies assessing the influence of HPV infection on sperm parameters and fertility outcomes in observational (case‐control, cross‐sectional, cohort) studies.

The WHO sperm parameters values were considered as reference values.^14^

Studies were excluded if the data were not analyzable; in vitro studies; if it was not possible to consider separated groups (HPV positive and negative) or if they did not clearly report data regarding sperm parameters or fertility outcomes. No language restriction was a priori applied.

### 2.3 Data extraction

For each eligible study, two independent investigators (MT and DP) extracted: name of the first author and year of publication, setting, sample size, mean age of the population, mean body mass index (BMI), HPV status and genotype, sperm parameters, and fertility outcomes. Data about matching and method (i.e. propensity score) were planned to be extracted between infected and controls, but no study included this information. Any inconsistencies were resolved by consensus with a third author (LS).

### 2.4 Outcomes

The primary outcomes considered regarded sperm parameters investigated as sperm count, volume, concentration, motility, vitality, morphology, ASAs, DNA fragmentation, chromatin damage and reproductive outcomes. All parameters were reported in the original papers as mean with standard deviations (SDs).

### 2.5 Assessment of study quality

Two independent authors (MT and DP) carried out the quality assessment of included studies’ using the Newcastle‐Ottawa Scale (NOS).^15^ The NOS assigns a maximum of 9 points based on three quality parameters: selection, comparability, and outcome.^16^ Any inconsistencies were resolved by consensus with a third author (LS).

### 2.6 Data synthesis and statistical analysis

All analyses were performed using the meta package inR.^17^ For all analyses, a two‐sided *p* value less than 0.05 was considered statistically significant. Studies were eligible for inclusion in the meta‐analysis if mean data and standard deviations were available for variables in both the HPV+ and HPV‐ groups. If studies had more than one outcome within the same domain (e.g., two outcomes relating to sperm volume), both the HPV+ and control groups were required to be independent to be included (e.g. the outcomes could not share the same control group).

For all analyses, a random effects meta‐analysis using the DerSimonian method^18^ was employed, with studies weighted on their inverse variance. Publication bias was assessed using the Egger's test.^19^ Heterogeneity was assessed using the I^2^ statistic.

#### 2.6.1 Sensitivity analyses

To determine the robustness of results, a sensitivity analysis was performed using the one‐study removed method.

#### 2.6.2 Credibility of evidence

The credibility of all results was classified according to the GRADE criteria, initially based on guidelines proposed by Schünemann et al.^20^

## 3 RESULTS

### 3.1 Literature search

**Figure 1:**
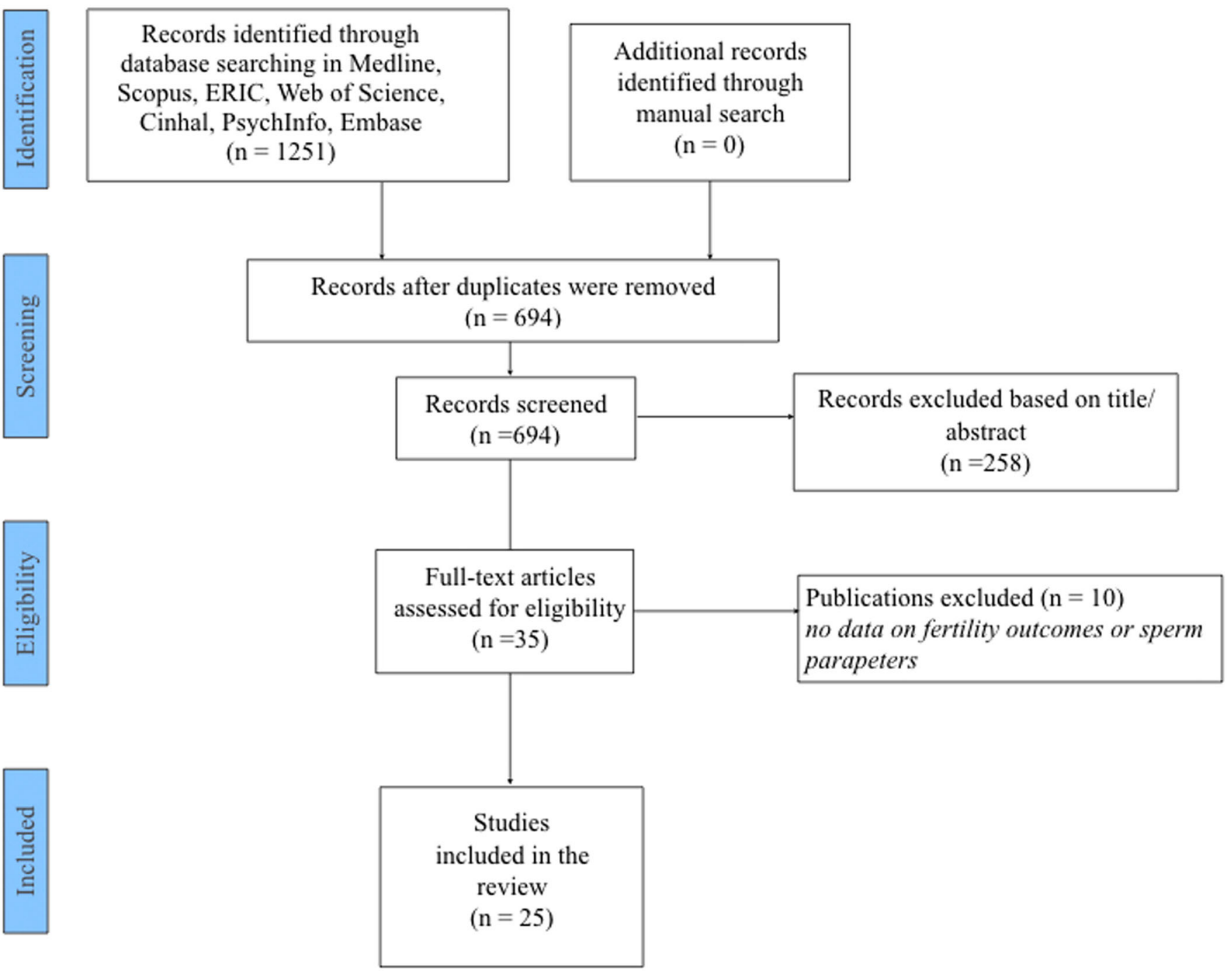
PRISMA flow chart.

As shown in Figure 1, 694 articles were initially screened and 35 full texts were retrieved. Among them, 25^21^, ^22^, ^23^, ^24^, ^25^, ^26^, ^27^, ^28^, ^29^, ^30^, ^31^, ^32^, ^33^, ^34^, ^35^, ^36^, ^37^, ^38^, ^39^, ^40^, ^41^, ^42^, ^43^, ^44^, ^45^ studies were finally included in the systemic review.

### 3.2 Descriptive findings and quality assessment

The 25 included studies were carried out mainly in Europe (*n* = 16), Asia (*n* = 6), South America (*n* = 2) and Middle‐East (*n* = 1). Overall, 6942 participants (range: 24–1173) were included having a mean age, for studies with available data, of 35 years (range: 31.16–39.2).

The median quality of the studies was 5.32 (range: 4–7), indicating an overall good quality of the studies, according to the NOS.

### 3.3 Influence of HPV infection on sperm parameters

Table 1 reports the main information and findings of sperm parameters of included studies. A total of 10 studies with 14 independent outcomes had enough data to be included in the meta‐analysis. A total of 10 studies (with 14 independent outcomes) examined sperm motility and sperm morphology, eight studies (nine outcomes) examined sperm concentration, five studies (ten independent outcomes) examined sperm volume, and four studies (eight independent outcomes) examined sperm pH.

**Table 1:** Main information and findings of sperm parameters of included studies.

Study				HPV positive						HPV negative					
	Country	Sample size	Detection	Count	Volume	Concentration	Motility	pH	Morphology	Count	Volume	Concentration	Motility	pH	Morphology
Boeri, 2018^22^	Italy	729	PCR	NA	3,0	10,9	16	NA	1,0	NA	3,0	12,4	22,0	NA	2,0
Cannarella, 2021^24^	Italy	40	PCR	67,4	NA	24,8	50,25	NA	18,5	73,0	NA	25,7	58,8	NA	20,5
Capra, 2022^25^	Italy	117	PCR	116,5	3	38,3	43,7	NA	4,4	123,5	2,8	49,6	46	NA	8,5
Damke, 2017^26^	Brazil	229	PCR	46	2,9	NA	42,4	7,8	NA	48	3,5	NA	49,8	7,7	NA
De Lima Bossi, 2019^27^	Brazil	25	PCR	NA	NA	62,3	65	NA	NA	NA	NA	65,9	46,6	NA	NA
Didelot‐rousseau, 2007^29^	France	62	PCR	3,2	NA	NA	NA	NA	NA	16	NA	NA	NA	NA	NA
Faja, 2024^31^	Italy	177	PCR	98	2,5	37,5	40	NA	10	108	3	42	45	NA	10
Foresta, 2010^32^	Italy	26	PCR e FISH	NA	2,6	53,5	36,2	7,7	32,6	177,1	0,8	NA	56,2	7,4	36,3
Foresta, 2010^32^	Italy	66	PCR e FISH	NA	2,8	48,5	38,4	7,6	31,8	178,4	2,5	NA	53,8	7,7	31,8
Foresta, 2010^32^	Italy	108	PCR e FISH	NA	2,9	30,0	33,9	7,7	32,9	102,9	3,0	NA	51,7	3,0	33,1
Foresta, 2010^32^	Italy	90	PCR e FISH	NA	2,5	60,5	55,5	7,6	33,5	176	2,6	NA	54,2	7,7	33,0
Garolla, 2012^33^	Italy	35	PCR e FISH	87,7	3,1	29,0	29,6	7,6	19,0	98,8	3,3	30,5	42,4	7,5	21,1
Garolla, 2013^34^	Italy	165	Inno‐Lipa, FISH	94,2	NA	32	29,0	NA	18,8	108,8	NA	34.6	47,8	NA	18,5
Garolla, 2015^35^	Italy	226	FISH	145,6	2,3	58,9	25,9	NA	16,2	131,9	2,7	52,2	34,3	NA	14,8
Jaworek, 2021^33^	Czech Republic	97	PCR	161,5	4,8	31,2	50	7,3	10,5	167,2	3	56,5	51,5	7.4	11
Jaworek, 2021^36^	Czech Republic	328	PCR	33	2	13	53	8	5	71,8	3	26	58,5	8	7
Lai,1997^37^	China	24	PCR	NA	NA	NA	40,4	NA	75	NA	NA	NA	62,7	NA	79,3
Luttmer, 2016^38^	The Netherlands	430	PCR	157,5	3,1	52,1	60,2	8,1	NA	189,3	3,4	57,5	57,9	8,1	NA
Moghimi, 2019^39^	Iran	70	PCR	NA	NA	51,38	23,50	NA	7,13	NA	NA	60,71	32,21	NA	15,18
Niakan, 2023^40^	Iran	140	PCR	NA	4,26	49,33	47,28	7,76	1,55	NA	3,91	58,96	50,72	7,69	2
Rintala, 2004^42^	Finland	65	PCR	297,1	3,7	96,5	54,2	7,37	NA	412,1	4,4	108,7	56,5	7,51	NA
Tongal, 2019^43^	Turkey	117	PCR	NA	NA	24	NA	NA	4	NA	NA	20	NA	NA	4
Yang, 2013^44^	China	615	PCR	NA	2,67	111,31	20,55	7,3	4,66	NA	2,65	120,96	29,11	7,26	8,15
Yang, 2013^44^	China	523	PCR	NA	2,31	114,42	32,25	7,03	8,51	NA	2,72	117,52	39,22	7,3	13,01

**Figure 2:**
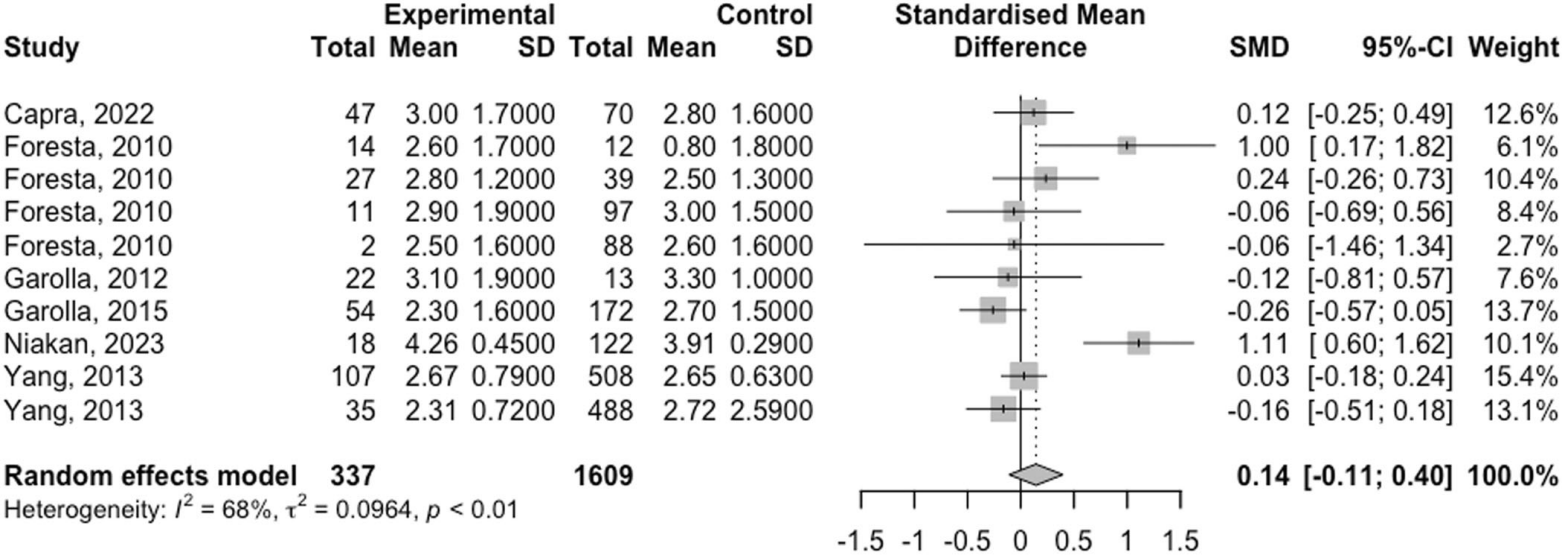
Sperm volume levels in HPV+ populations vs. HPV‐ controls.

**Figure 3:**
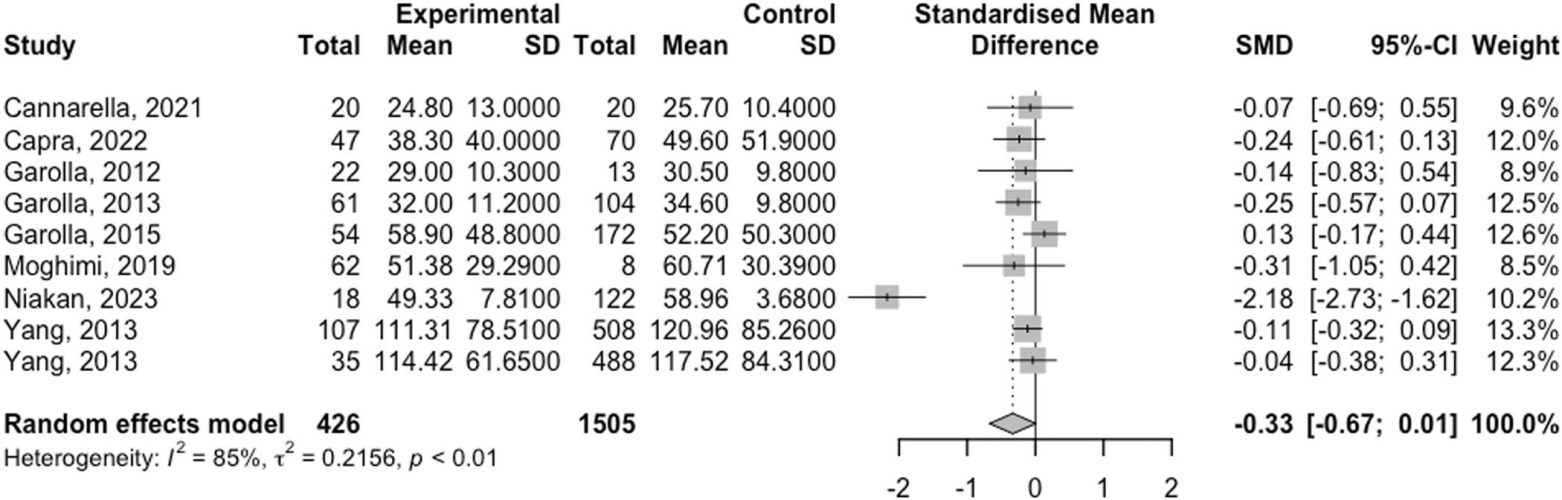
Sperm concentration levels in HPV+ populations vs. HPV‐ controls.

**Figure 4:**
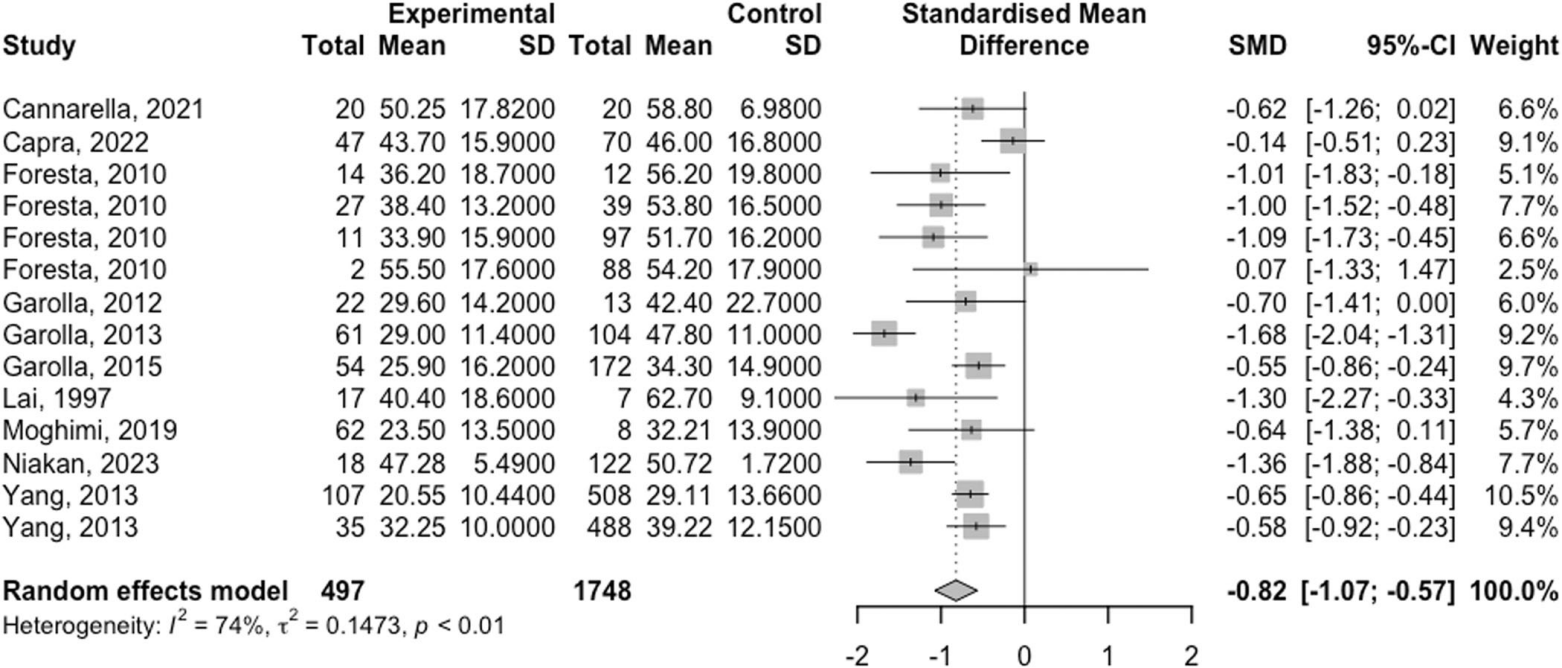
Sperm motility levels in HPV+ populations vs. HPV‐ controls.

**Figure 5:**
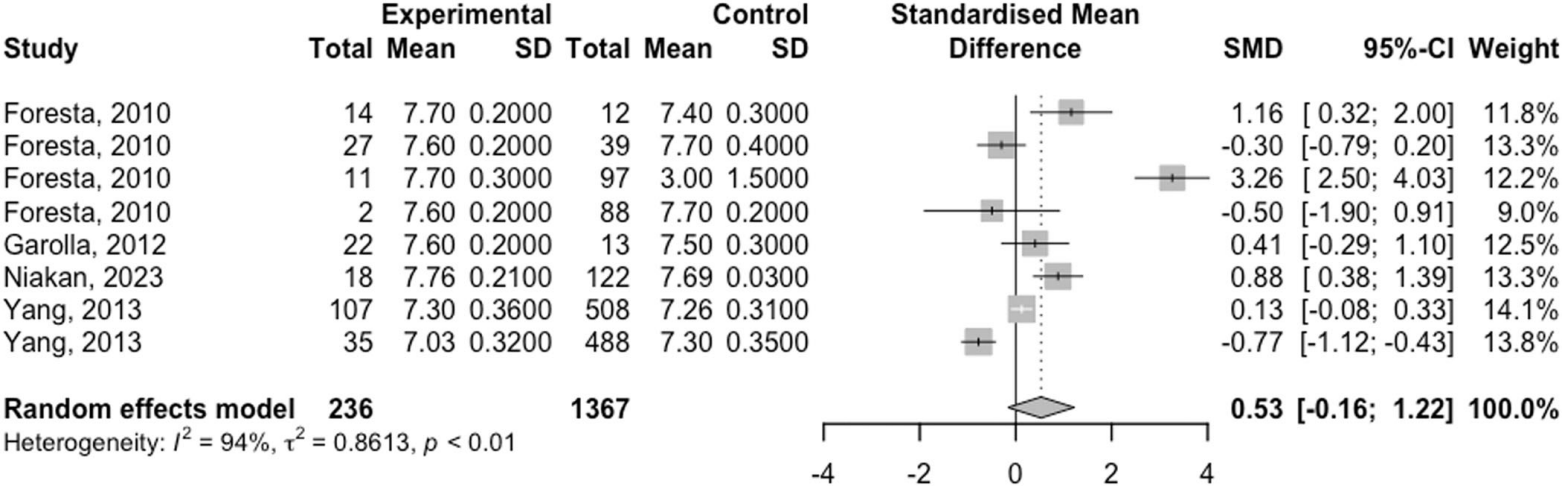
Sperm pH levels in HPV+ populations vs. HPV‐ controls.

**Figure 6:**
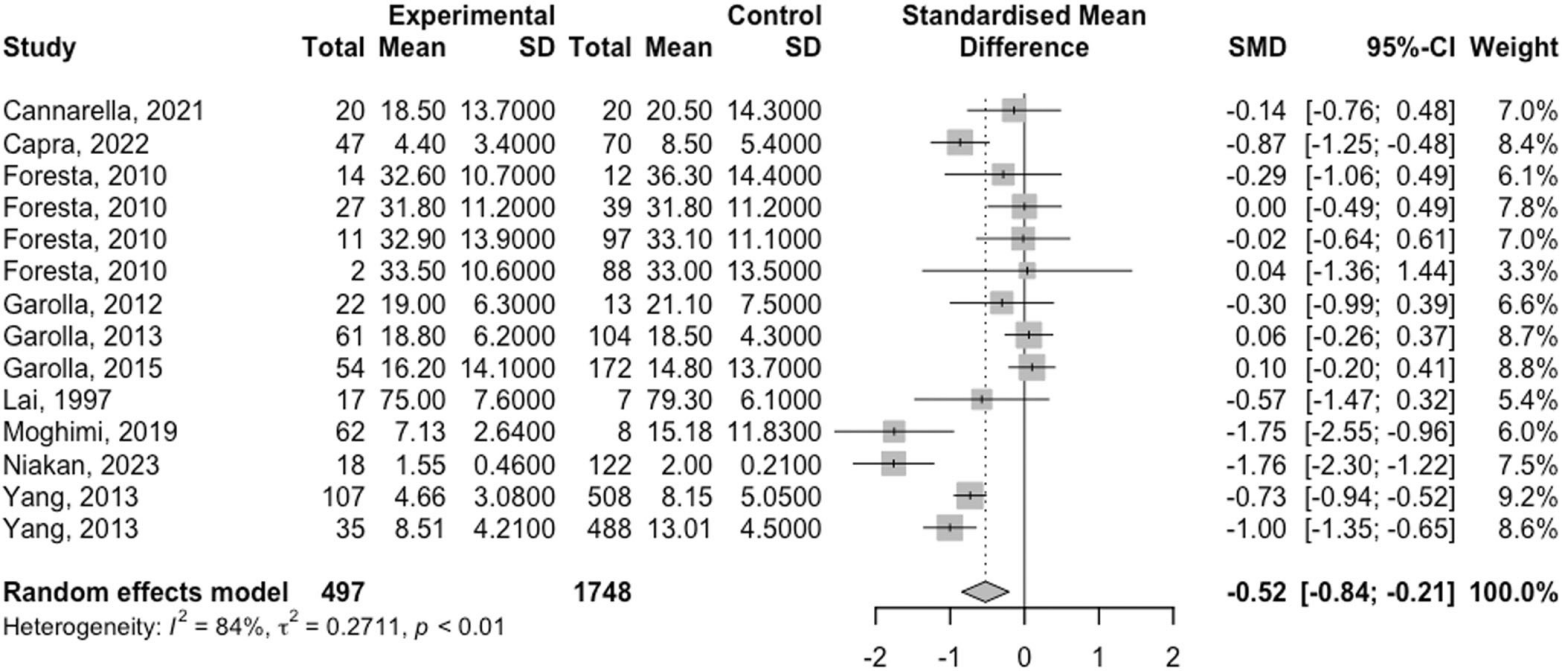
Sperm morphology levels in HPV+ populations vs. HPV‐ controls.

Full meta‐analysis results can be found in Table 2 and Figures 2, 3, 4, 5, 6. Sperm morphology was significantly lower in HPV+ groups versus HPV‐ control groups (SMD = −0.52 95% CI −0.84; −0.21; *p* = 0.001). Sperm motility was also significantly lower in HPV+ groups when compared to HPV‐controls (SMD = −0.82 95% CI −1.07; −0.57; *p* = <0.001). Sperm volume, concentration, and pH were not significantly different. Sensitivity analyses revealed that the removal of any one study did not affect the significance of any outcome.

**Table 2:** Full meta‐analysis results.

Outcome	*k studies (n* outcomes)	Meta‐analysis		Heterogeneity	Publication bias	Level of certainty
		SMD (95% CI)	*p* value	*I* ^2^	Egger's *p* value	
Sperm volume	5 (10)	0.14 (−0.11; 0.40)	0.266	67.9	0.30	NS
Sperm concentration	9 (8)	−0.33 (−0.67; 0.01)	0.057	85.3	NA	NS
Sperm motility	10 (14)	−0.82 (−1.07; −0.57)	<0.001	73.6	0.54	Moderate^a^
Sperm pH	4 (8)	0.53 (−0.16; 1.22)	0.133	93.6	NA	NS
Sperm morphology	9 (14)	−0.52 (−0.84; −0.21)	0.001	84.2	0.96	Moderate^a^

Abbreviations: SMD, standardized mean difference; NA, not applicable due to number of studies under 10; NS, not significant

^a^ Downgraded due to high heterogeneity.

### 3.4 Fertility and reproductive outcomes

Table 3 reports the main information and findings on fertility and reproductive outcomes of included studies. Female HPV infection was associated with lower rates of implantation, biochemical pregnancy, and clinical pregnancy as well as with a slightly higher frequency of biochemical miscarriage and clinical miscarriage.^21^ The presence of HPV infection in sperm impacts negatively on assisted reproduction with a reduction of clinical pregnancies^28^, ^30^, ^45^ and increase in pregnancy loss.^23^, ^41^

**Table 3:** Main information and findings on fertility and reproductive outcome of included studies.

Author and date	Aim	Study type	Sample size and characteristics	Outcome measures	Findings
Bai, 2024^21^	To assess the effects of intrauterine HPV on ART outcomes in infertile couples	Cohort Study	4153 infertile women undergoing IVF or ICSI treatment	Pregnancy and clinical miscarriage rates in the transfer cycle underwent HPV detection	Uterine cavity HPV infection resulted in lower rates of ongoing, implantation, biochemical pregnancy, and clinical pregnancy compared with HPV negative group. The infertile female with positive HPV also had a slightly higher frequency of biochemical miscarriage and clinical miscarriage.
Busnelli, 2023^23^	To assess the association between HPV sperm infection and idiopathic recurrent pregnancy loss	Case‐control	117 cases were men of couples affected by first trimester idiopathic recurrent pregnancy loss. 84 controls were men of couples with proven fertility	The association between HPV sperm infection and recurrent pregnancy lost	The prevalence of HPV sperm infection was significantly higher in couples affected by recurrent pregnancy lost than in their controls. The semen sample was infected by HPV in approximately 1 out of 5 patients.
Depuydt, 2019^28^	To study the influence of HPV in sperm of male partners of women undergoing IUI	Cohort study	7320 infertile couples undergoing 1753 IUI cycles	Biochemical and clinical pregnancy rate in IUI	Women inseminated with HPV positive sperm had 4 times fewer clinical pregnancies compared with women who had HPV negative partners.
Depuydt, 2021^30^	To determine the sperm DNA fragmentation and HPV infection impact on clinical pregnancies in women undergoing IUI	Cohort study	161 infertile couples going through 209 cycles of IUI	Clinical pregnancies in women undergoing IUI	Both the sperm DNA fragmentation and HPV infection negatively impacted IUI. When HPV was present in sperm, no clinical pregnancies were observed.
Perino, 2011^41^	To assess the relationship between HPV infection in and ART outcome	Cohort study	Of 199 couples, 33 underwent oocyte insemination, and 166 intracytoplasmic sperm injection.	Clinical pregnancy rate and pregnancy loss rate	A highly statistically significant correlation between pregnancy loss rate and positive HPV DNA testing in the male was observed. No statistically significant differences in term of pregnancy rate.
Zhang, 2019^45^	To demonstrate the association of HPV infection and pregnancy, delivery and neonatal outcome in women undergoing IUI	Cohort study	329 women undergoing IUI	Clinical pregnancy rate, miscarriage rate, live birth rate, gestational weeks, neonatal weight, sex, and delivery mode	HPV infection could reduce clinical pregnancy rate and live birth rate significantly of women undergoing IUI. There was no significant difference for miscarriage rate. Gestational weeks showed significant difference between two groups while no difference was noted in neonatal weight, sex or delivery mode.

Abbreviations: ART, assisted reproduction technologies; HPV, Human Papillomavirus; ICSI, intracytoplasmic sperm injection; IVF, In vitro fertilization.

## 4 DISCUSSION

The findings of our systematic review and meta‐analysis suggest a role of HPV infection in men both in impairing sperm parameters and in affecting pregnancy outcomes. In terms of sperm parameters, sperm morphology and motility were lower in HPV positive males compared to HPV negative. These results are in line with one previous review focusing on sperm quality.^46^ However, sperm volume, concentration, and pH did not show significant differences between HPV infected males and those not infected. Possible mechanisms hypothesized for explaining impairment in parameters is likely owing to the presence of HPV itself and the highest presence of ASA.^34^ Indeed, previous literature has demonstrated an interaction between HPV with sperm receptors localized in the equatorial region of the sperm head.^47^ Moreover, the presence of HPV in the sperm is frequently associated with ASAs in infertile patients.^34^, ^38^ Moreover, ASAs may affect male fertility through sperm agglutination and impaired cervical mucus penetration, complement‐mediated sperm injury through the female genital tract, and interference with gametes interaction.^34^ Considering that autoimmune infertility is often treated by assisted reproduction techniques (ART) and that these procedures can be ineffective in the presence of HPV sperm infection, it is recommended screening for HPV in all asymptomatic subjects with ASAs.

Studies assessing pregnancy and ART outcomes related to HPV infection are still too limited to perform any statistical analysis, but important considerations can be drawn from existing literature. Indeed, Perino et al. found a significant increased risk of pregnancy loss in couples who underwent ART when HPV was present in sperm.^41^ Another study reported the reduction in both natural and assisted cumulative pregnancy rate and an increase in miscarriage rate in association with the presence of HPV at sperm level.^35^ Interestingly, women underwent intra uterine insemination with HPV positive sperm showed 4 times fewer clinical pregnancies compared with those with negative partners.^28^ Recently, another study revealed that the prevalence of HPV sperm infection is significantly higher in couples affected by recurrent pregnancy loss compared to controls.^23^ Similar results have been observed also in cases of positive women. Indeed, women with HPV infection are associated with lower rates of implantation, biochemical pregnancy, and clinical pregnancy as well as with a slightly higher frequency of biochemical miscarriage and clinical miscarriage.^21^, ^48^ Again, in one study HPV positive women which underwent intrauterine insemination had six times less pregnancies than those wo were HPV negative.^49^

Several mechanisms have been hypothesized by which HPV infection can lead to infertility and its negative impact on the success of spontaneous and assisted reproduction could be related to the alteration of sperm parameters, induction of DNA damage, and genomic instability.^50^ Moreover, in vitro studies found that HPV‐transfected trophoblast cells have an increased rate of stage‐specific maturation arrest and apoptosis and a reduced placental invasion into the uterine wall compared with control cells.^35^ Moreover, HPV can compromise trophoblast engraftment and embryo development, leading to pregnancy loss.^9^ Finally, anti‐HPV immune response could cause infertility rejecting the HPV‐infected embryo caused by maternal graft‐versus‐host disease against HPV infected fetus.^9^ Importantly, host immune responses and vaginal microbiome have a natural role in HPV infection, although HPV use different immune evasion mechanisms to limit antiviral activity of immune response inducing tolerance in the host's immune system and reducing the natural clearance.^51^

Independent from the mechanism, it is urgent to find integrated and multidisciplinary solutions which consider the complexity of reproductive care. Although it is not possible from our results to draw final conclusions, we can make some recommendations.

First, HPV DNA testing, also considering its low cost compared to a failed ART procedure, in male partners of infertile couples, even if asymptomatic, could be useful to find this possible infertility cause and, thus, find the best solution. Considering that the estimated clearance for the virus in sperm is >60% at 6 months, in the case of young couples there is the possibility for waiting for spontaneous healing to restore normal sperm parameters and aim for spontaneous pregnancy or to improve the ART outcomes. Instead, in case of aged couples with no possibility for waiting for spontaneous clearance, specific enzymatic washing procedure of infected semen have been shown to be effective for HPV removal.^33^, ^52^ Moreover, HPV vaccination has been suggested, showing promising findings, not only in preventing clinical manifestations but also in speeding up the clearance.^53^, ^54^ In particular, the nonavalent HPV vaccine seems to offer wider protection especially in men with positive HPV partners, which would play a role in the transmission of the infection and relapse. However, considering that a significant reduction in HPV related infections can be achieved only through vaccination coverage above 80%, extensive vaccination programs should be planned also in a public health economically viable approach.^55^

In addition to these considerations, counseling should be tailored for infected couples paying particular attention to the hygiene of the reproductive tract and of the hands, strict use of personal underwear and personal towels, a complete abstinence from oral and anal sex, smoking reduction or cessation, the practice of protected intercourse only, and, in the case of HPV‐related lesions, the treatment and the monitoring of the genital area.^56^

Findings from this meta‐analysis must be considered in light of its limitations: I) not all studies considered all sperm parameters; II) some important confounding factors, such as male age and environmental exposures were not always noted; and III) despite the clear negative impact on sperm and pregnancy outcomes, the exact pathophysiological mechanisms are not fully understood.

In conclusion, the present work identified associations between HPV and sperm parameters and reproductive outcomes, which has the potential to lead to a decreased couple fertility, increased risk of pregnancy loss, re‐infection and increased treatment costs. Counseling and vaccination are two pillars for the management of infected couples, However, it is urgent for reproductive specialists, health professionals, researchers and policy makers to collaborate to develop the best models of HPV management. Such models should be tailored to male and female infected patients to prevent infection transmission, reinfection, speed up clearance, and ultimately to improve and increase reproductive outcomes.

## FUNDS

None to declare.

## AUTHOR CONTRIBUTIONS

**Andrea Garolla**: Conceptualization; Supervision. **Silvia Mereu**: Investigation; Writing—original draft. **Damiano Pizzol**: Writing—review and editing; Investigation. **Dong Keon Yon**: Writing—review and editing; Methodology. **Masoud Rahmati**: Visualization; Formal analysis. **Pinar Soysal**: Writing—original draft; Data curation. **Petre Cristian Ilie**: Methodology; Data curation. **Alessandro Bertoldo**: Data curation; Investigation; Formal analysis. **Mike Trott**: Software; Data curation; Visualization. **Lee Smith**: Writing—review and editing; Funding acquisition.

## CONFLICT OF INTEREST STATEMENT

The authors declare no conflict of interest.

## TRANSPARENCY STATEMENT

The lead author Damiano Pizzol affirms that this manuscript is an honest, accurate, and transparent account of the study being reported; that no important aspects of the study have been omitted; and that any discrepancies from the study as planned (and, if relevant, registered) have been explained.

## Data Availability

The data that support the findings of this study are available from the corresponding author upon reasonable request. The data are available upon request. Available from DP (damianopizzol8@gmail.com)

## References

[1] Bruni L , Albero G , Rowley J , et al. Global and regional estimates of genital human papillomavirus prevalence among men: a systematic review and meta‐analysis. Lancet Glob Health. 2023;11(9):e1345‐e1362. 37591583 10.1016/S2214-109X(23)00305-4 PMC10447222

[2] Working Group on the Evaluation of Carcinogenic Risks to Humans. Human papillomaviruses. IARC Monogr Eval Carcinog Risks Hum. 2007;90:1‐636. 18354839 PMC4781057

[3] Boda D , Docea AO , Calina D , et al. Human papilloma virus: apprehending the link with carcinogenesis and unveiling new research avenues (Review). Int J Oncol. 2018;52(3):637–655. 29393378 10.3892/ijo.2018.4256 PMC5807043

[4] Kombe Kombe AJ , Li B , Zahid A , et al. Epidemiology and burden of human papillomavirus and related diseases, molecular pathogenesis, and vaccine evaluation. Front Public Health. 2021;8:552028. 33553082 10.3389/fpubh.2020.552028 PMC7855977

[5] de Martel C , Georges D , Bray F , Ferlay J , Clifford GM . Global burden of cancer attributable to infections in 2018: a worldwide incidence analysis. Lancet Glob Health. 2020;8(2):e180‐e190. 31862245 10.1016/S2214-109X(19)30488-7

[6] Giuliano AR , Anic G , Nyitray AG . Epidemiology and pathology of HPV disease in males. Gynecol Oncol. 2010;117(2 suppl):S15‐S19. 20138345 10.1016/j.ygyno.2010.01.026 PMC4254924

[7] WHO Guideline for Screening and Treatment of Cervical Pre‐Cancer Lesions for Cervical Cancer Prevention [Internet]. 2nd ed. World Health Organization; 2021. PMID: 34 314129. 34314129

[8] CDC Centers for Diseases Control and Prevention . HPV and Men. https://www.cdc.gov/std/hpv/stdfact-hpv-and-men.htm (Accessed January 2024).

[9] Isaguliants M , Krasnyak S , Smirnova O , Colonna V , Apolikhin O , Buonaguro FM . Genetic instability and anti‐HPV immune response as drivers of infertility associated with HPV infection. Infect Agent Cancer. 2021;16(1):29. 33971936 10.1186/s13027-021-00368-1 PMC8111735

[10] Cao X , Wei R , Zhang X , Zhou J , Lou J , Cui Y . Impact of human papillomavirus infection in semen on sperm progressive motility in infertile men: a systematic review and meta‐analysis. Reprod Biol Endocrinol. 2020;18(1):38. 32381092 10.1186/s12958-020-00604-0 PMC7203819

[11] Moreno‐Sepulveda J , Rajmil O . Seminal human papillomavirus infection and reproduction: a systematic review and meta‐analysis. Andrology. 2021;9(2):478‐502. 33220146 10.1111/andr.12948

[12] Liberati A , Altman DG , Tetzlaff J , et al. The PRISMA statement for reporting systematic reviews and meta‐analyses of studies that evaluate health care interventions: explanation and elaboration. PLoS Med. 2009;6(7):e1000100. 19621070 10.1371/journal.pmed.1000100 PMC2707010

[13] Stroup DF . Meta‐analysis of observational studies in epidemiology: a proposal for reporting. Meta‐analysis Of Observational Studies in Epidemiology (MOOSE) group. JAMA. 2000;283:2008‐2012. 10789670 10.1001/jama.283.15.2008

[14] Cooper TG , Noonan E , von Eckardstein S , et al. World health organization reference values for human semen characteristics. Hum Reprod Update. 2010;16(3):231‐245. 19934213 10.1093/humupd/dmp048

[15] Wells GA , Shea B , O'Connell D , et al. The Newcastle‐Ottawa Scale (NOS) for assessing the quality if nonrandomized studies in meta‐analyses. http://wwwohrica/programs/clinical_epidemiology/oxfordasp (Accessed January 2024).

[16] Luchini C , Stubbs B , Solmi M , et al Assessing the quality of studies in meta‐analysis: advantages and limitations of the Newcastle Ottawa Scale. World J Meta‐Anal. 2017;5:1‐48.

[17] Balduzzi S , Rücker G , Schwarzer G . How to perform a meta‐analysis with R: a practical tutorial. Evid Based Ment Health. 2019;22:153‐160. 31563865 10.1136/ebmental-2019-300117 PMC10231495

[18] DerSimonian R , Laird N . Meta‐analysis in clinical trials. Control Clin Trials. 1986;7(3):177‐188. 3802833 10.1016/0197-2456(86)90046-2

[19] Egger M , Smith GD , Schneider M , Minder C , et al. Bias in meta‐analysis detected by a simple, graphical test. BMJ. 1997;315:629‐634. 9310563 10.1136/bmj.315.7109.629 PMC2127453

[20] Guyatt GH , Oxman AD , Vist GE , et al. GRADE: an emerging consensus on rating quality of evidence and strength of recommendations. BMJ. 2008;336:924‐926. 18436948 10.1136/bmj.39489.470347.AD PMC2335261

[21] Bai M , Sun D , Shu J , et al. Assisted reproductive technology treatment failure and the detection of intrauterine HPV through spent embryo transfer media sample. J Med Virol. 2024;96(3):e29468. 38415499 10.1002/jmv.29468

[22] Boeri L , Capogrosso P , Ventimiglia E , et al. High‐risk human papillomavirus in semen is associated with poor sperm progressive motility and a high sperm DNA fragmentation index in infertile men. Hum Reprod. 2019;34(2):209‐217. 30517657 10.1093/humrep/dey348

[23] Busnelli A , Garolla A , Tersigni C , et al. Sperm human papillomavirus infection and risk of idiopathic recurrent pregnancy loss: insights from a multicenter case‐control study. Fertil Steril. 2023;119(3):410‐418. 36493870 10.1016/j.fertnstert.2022.12.002

[24] Cannarella R , Aversa A , Condorelli RA , et al. Impact of seminal low‐risk human papillomavirus infection on sperm parameters of adult men. Aging Male. 2022;25(1):17‐22. 34978266 10.1080/13685538.2021.2023126

[25] Capra G , Notari T , Buttà M , et al. Human papillomavirus (HPV) infection and its impact on male infertility. Life. 2022;12(11):1919. 36431054 10.3390/life12111919 PMC9697777

[26] Damke E , Kurscheidt FA , Balani VA , et al. Male partners of infertile couples with seminal infections of human papillomavirus have impaired fertility parameters. BioMed Res Int. 2017;2017:4684629. 28835893 10.1155/2017/4684629 PMC5556607

[27] Bossi RL , Valadares JBF , Puerto HLD , et al. Prevalence of human papillomavirus (HPV) in the semen of patients submitted to assisted reproductive technology treatment in a private clinic in Brazil. JBRA Assist Reprod. 2019;23(3):205‐209. 30875170 10.5935/1518-0557.20190009 PMC6724393

[28] Depuydt CE , Donders GGG , Verstraete L , et al. Infectious human papillomavirus virions in semen reduce clinical pregnancy rates in women undergoing intrauterine insemination. Fertil Steril. 2019;111(6):1135‐1144. 31005311 10.1016/j.fertnstert.2019.02.002

[29] Didelot‐Rousseau MN , Diafouka F , Yayo E , Kouadio LP , Monnet D , Segondy M . HPV seminal shedding among men seeking fertility evaluation in Abidjan, Ivory Coast. J Clin Virol. 2007;39(2):153‐155. 17442614 10.1016/j.jcv.2007.03.003

[30] Depuydt C , Donders G , Verstraete L , et al. Negative impact of elevated DNA fragmentation and human papillomavirus (HPV) presence in sperm on the outcome of Intra‐Uterine insemination (IUI). J Clin Med. 2021;10(4):717. 33670283 10.3390/jcm10040717 PMC7917808

[31] Faja F , Pallotti F , Bianchini S , et al. Molecular study of the presence and transcriptional activity of HPV in semen. J Endocrinol Investig. 2024;47(3):557‐570. 37584897 10.1007/s40618-023-02167-4 PMC10904563

[32] Foresta C , Pizzol D , Moretti A , Barzon L , Palù G , Garolla A . Clinical and prognostic significance of human papillomavirus DNA in the sperm or exfoliated cells of infertile patients and subjects with risk factors. Fertil Steril. 2010;94(5):1723‐1727. 20056213 10.1016/j.fertnstert.2009.11.012

[33] Garolla A , Lenzi A , Palu G , et al. Human papillomavirus sperm infection and assisted reproduction: a dangerous hazard with a possible safe solution. Hum Reprod. 2012;27(4):967‐973. 22313870 10.1093/humrep/des009

[34] Garolla A , Pizzol D , Bertoldo A , De Toni L , Barzon L , Foresta C . Association, prevalence, and clearance of human papillomavirus and antisperm antibodies in infected semen samples from infertile patients. Fertil Steril. 2013;99(1):125‐131.e2. 23043686 10.1016/j.fertnstert.2012.09.006

[35] Garolla A , Engl B , Pizzol D , et al. Spontaneous fertility and in vitro fertilization outcome: new evidence of human papillomavirus sperm infection. Fertil Steril. 2016;105(1):65‐72.e1. 26453270 10.1016/j.fertnstert.2015.09.018

[36] Jaworek H , Koudelakova V , Oborna I , et al. Impact of human papillomavirus infection on semen parameters and reproductive outcomes. Reprod Biol Endocrinol. 2021;19(1):156. 34627284 10.1186/s12958-021-00840-y PMC8501609

[37] Lai YM , Lee JF , Huang HY , Soong YK , Yang FP , Pao CC . The effect of human papillomavirus infection on sperm cell motility. Fertil Steril. 1997;67(6):1152‐1155. 9176459 10.1016/s0015-0282(97)81454-9

[38] Luttmer R , Dijkstra MG , Snijders PJ , et al. Presence of human papillomavirus in semen in relation to semen quality. Hum Reprod. 2016;31(2):280‐286. 26724799 10.1093/humrep/dev317

[39] Moghimi M , Zabihi‐Mahmoodabadi S , Kheirkhah‐Vakilabad A , Kargar Z . Significant correlation between High‐Risk HPV DNA in semen and impairment of sperm quality in infertile men. Int J Fertil Steril. 2019;12(4):306‐309. 30291691 10.22074/ijfs.2019.5421 PMC6186290

[40] Niakan S , Faghihloo E , Shams Mofarahe Z , et al. Evaluation of human papillomavirus in the semen of infertile men and its relationship with semen quality. Arch Clin Infect Dis. 2023;18(4):e139376.

[41] Perino A , Giovannelli L , Schillaci R , et al. Human papillomavirus infection in couples undergoing in vitro fertilization procedures: impact on reproductive outcomes. Fertil Steril. 2011;95(5):1845‐1848. 21167483 10.1016/j.fertnstert.2010.11.047

[42] Rintala MAM , Greénman SE , Pöllänen PP , Suominen JJO , Syrjänen SM . Detection of high‐risk HPV DNA in semen and its association with the quality of semen. Int J STD AIDS. 2004;15(11):740‐743. 15537460 10.1258/0956462042395122

[43] Tangal S , Tasci Y , Pabuccu EG , Caglar GS , Haliloglu AH , Yararbas K . DNA fragmentation index and human papilloma virus in males with previous assisted reproductive technology failures. Türk Üroloji Dergisi/Turk J Urol. 2019;45(1):12‐16. 10.5152/tud.2018.96393 PMC6342580 29975635

[44] Yang Y , Jia CW , Ma YM , Zhou LY , Wang SY . Correlation between HPV sperm infection and Male infertility. Asian J Androl. 2013;15(4):529‐532. 23603919 10.1038/aja.2013.36 PMC3739240

[45] Zhang B , Xiao Y , Wang M , et al Human papillomaviruse infection in women undergoing artificial insemination by donor: a retrospective analysis. Int J Clin Exp Med. 2019;12(3):2980‐2985.

[46] Wang S , Liu L , Zhang A , Song Y , Kang J , Liu X . Association between human papillomavirus infection and sperm quality: a systematic review and a meta‐analysis. Andrologia. 2021;53(5):e14034. 33666259 10.1111/and.14034

[47] Foresta C , Patassini C , Bertoldo A , et al. Mechanism of human papillomavirus binding to human spermatozoa and fertilizing ability of infected spermatozoa. PLoS One. 2011;6(3):e15036. 21408100 10.1371/journal.pone.0015036 PMC3051064

[48] Spandorfer SD , Bongiovanni AM , Fasioulotis S , Rosenwaks Z , Ledger WJ , Witkin SS . Prevalence of cervical human papillomavirus in women undergoing in vitro fertilization and association with outcome. Fertil Steril. 2006;86(3):765‐767. 16782096 10.1016/j.fertnstert.2006.01.051

[49] Depuydt CE , Verstraete L , Berth M , et al. Human papillomavirus positivity in women undergoing intrauterine insemination has a negative effect on pregnancy rates. Gynecol Obstet Investig. 2016;81(1):41‐46. 26160018 10.1159/000434749

[50] Sucato A , Buttà M , Bosco L , Di Gregorio L , Perino A , Capra G . Human papillomavirus and Male infertility: what do we know? Int J Mol Sci. 2023;24(24):17562. 38139389 10.3390/ijms242417562 PMC10744208

[51] Ntuli L , Mtshali A , Mzobe G , Liebenberg LJ , Ngcapu S . Role of immunity and vaginal microbiome in clearance and persistence of human papillomavirus infection. Front Cell Infect Microbiol. 2022;12:927131. 35873158 10.3389/fcimb.2022.927131 PMC9301195

[52] De Toni L , Cosci I , Carosso A , et al. Hyaluronidase‐based swim‐up for semen selection in patients with human papillomavirus semen infection. Biol Reprod. 2021;104(1):211‐222. 33164043 10.1093/biolre/ioaa173

[53] Muscianisi F , Foresta C , Garolla A . Role of HPV vaccination for prevention of male infertility. Minerva Endocrinol. 2022;47(1):70‐76. 10.23736/S2724-6507.22.03667-3 35166470

[54] WHO Efficacy and safety of therapeutic HPV vaccines to treat CIN 2/CIN 3 lesions: a systematic review and meta‐analysis of phase II/III clinical trials. https://www.iarc.who.int/news-events/efficacy-and-safety-of-therapeutic-hpv-vaccines-to-treat-cin-2-cin-3-lesions-a-systematic-review-and-meta-analysis-of-phase-ii-iii-clinical-trials/ (Accessed January 2024). 10.1136/bmjopen-2022-069616 PMC10603536 37879679

[55] Bosco L , Serra N , Fasciana T , et al. Potential impact of a nonavalent anti HPV vaccine in Italian men with and without clinical manifestations. Sci Rep. 2021;11(1):4096. 33603082 10.1038/s41598-021-83639-6 PMC7892856

[56] Garolla A , Pizzol D , Vasoin F , Barzon L , Bertoldo A , Foresta C . Counseling reduces HPV persistence in coinfected couples. J Sex Med. 2014;11(1):127‐135. 24165376 10.1111/jsm.12358

